# Supplementary material for: p53 restoration in small cell lung cancer identifies a latent cyclophilin-dependent necrosis mechanism
Source: Nat Commun. 2023 Jul 21;14:4403. doi: 10.1038/s41467-023-40161-9 (PMC10362054; doi:10.1038/s41467-023-40161-9)
Supplement: Supplementary file 1 — Supplementary Info [file 41467_2023_40161_MOESM1_ESM.pdf]

p53 restoration in small cell lung cancer identifies a latent cyclophilin-dependent necrosis mechanism

Jonuelle Acosta[1,2], Qinglan Li[1,], Nelson F. Freeburg[1,2,], Nivitha Murali[1], Alexandra Indeglia[3], Grant P. Grothusen[1,2], Michelle Cicchini[1], Hung Mai[1], Amy C. Gladstein[1,2], Keren M. Adler[1,2], Katherine R. Doerig[1,2], Jinyang Li[1], Miguel Ruiz-Torres[1], Kimberly L. Manning[1], Ben Z. Stanger[2,4], Luca Busino[1,2,4], Maureen Murphy[2,5], Liling Wan[1,2,4], David M. Feldser[1,2,4,*]

[1]Department of Cancer Biology, Perelman School of Medicine, University of Pennsylvania, Philadelphia, PA, USA
[2]Cell and Molecular Biology Graduate Group, Perelman School of Medicine, University of Pennsylvania, Philadelphia, PA, USA
[3]Biochemistry and Molecular Biophysics Graduate Group, Perelman School of Medicine, University of Pennsylvania, Philadelphia, PA, USA
[4]Abramson Family Cancer Research Institute, University of Pennsylvania, Philadelphia, PA, USA
[5]Program in Molecular and Cellular Oncogenesis, The Wistar Institute, Philadelphia, PA, USA
*Correspondence: dfeldser@upenn.edu

Supplementary Figures 1-18

Supplementary Table 1

Supplementary Figure 1

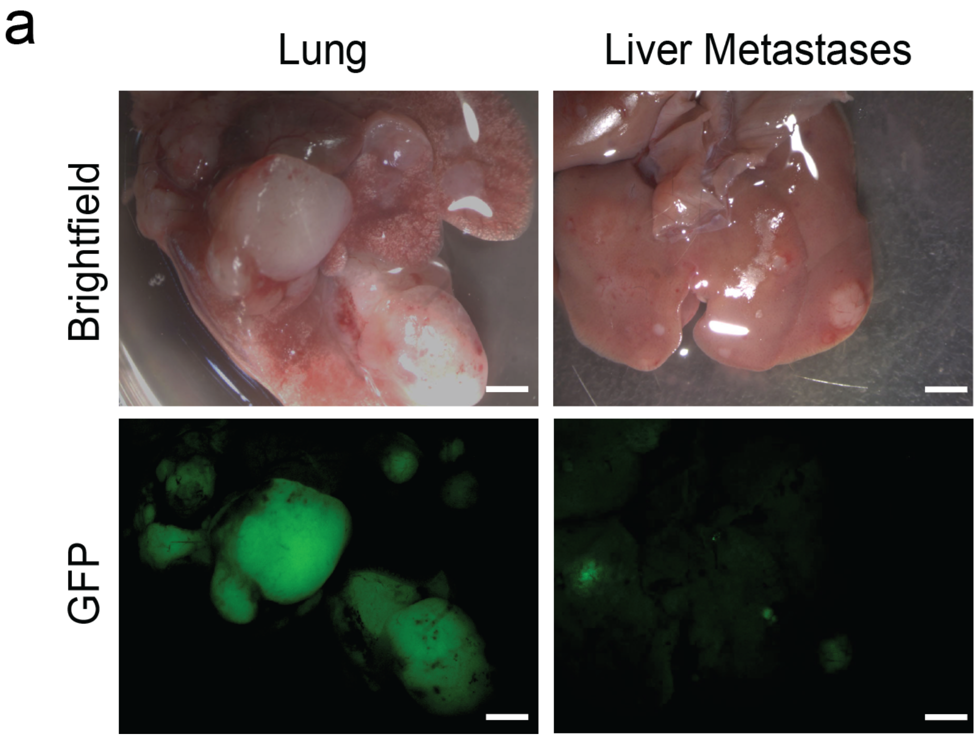

Supplementary Fig. 1. *RP^R^R2* animals succumb to rare *RP^TR^R2* GFP+ cells that escape tamoxifen-induced p53 restoration. (**A**) Brightfield and fluorescent micrographs of lungs and liver metastases from *RP^R^R2* mouse treated with tamoxifen for 14 weeks. Scale bars: 4.4mm.

Supplementary Figure 2

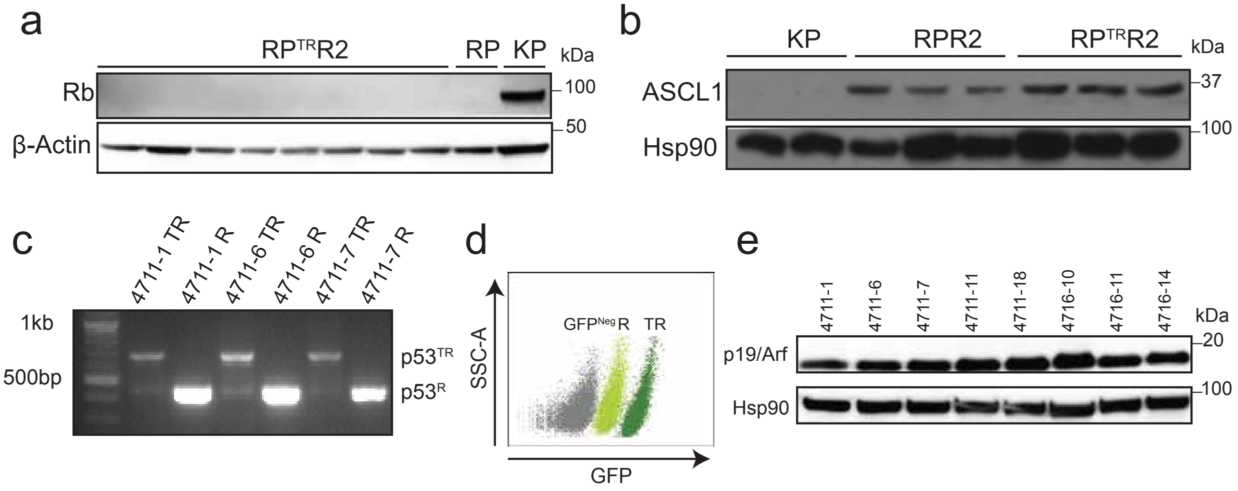

Supplementary Fig. 2. $RP^{TR}R2$ cell lines recapitulate features of SCLC and express p19/Arf. (**A**) Immunoblot analysis for Rb in *$RP^{TR}R2$* tumor-derived cell lines. *RP* and *KP* cell lines used as negative and positive controls for Rb expression, respectively. β-actin used as loading control. (**B**) Immunoblot analysis for expression of the neuroendocrine marker, ASCL1, in *$RP^{TR}R2$* tumor-derived cell lines. *KP* and *RPR2* cell lines used as negative and positive controls for ASCL1 expression, respectively. Hsp90 used as loading control. (**C**) PCR-based detection of *$p53^{TR}$* and *$p53^{R}$* alleles in *$RP^{TR}R2$* cell lines (n=3) after 24hrs of 4-OHT treatment. (**D**) Detection of GFP reporter expression from *TR* alleles in *$RP^{TR}R2$* cell lines by flow cytometry analysis. *KP* cell line used as GFP^{neg} control. Representative of n=8 cell lines.(**E**) Immunoblot analysis for p19/Arf in *$RP^{TR}R2$* tumor-derived cell lines. Hsp90 is loading control. Source data are provided as a Source Data file.

Supplementary Figure 3

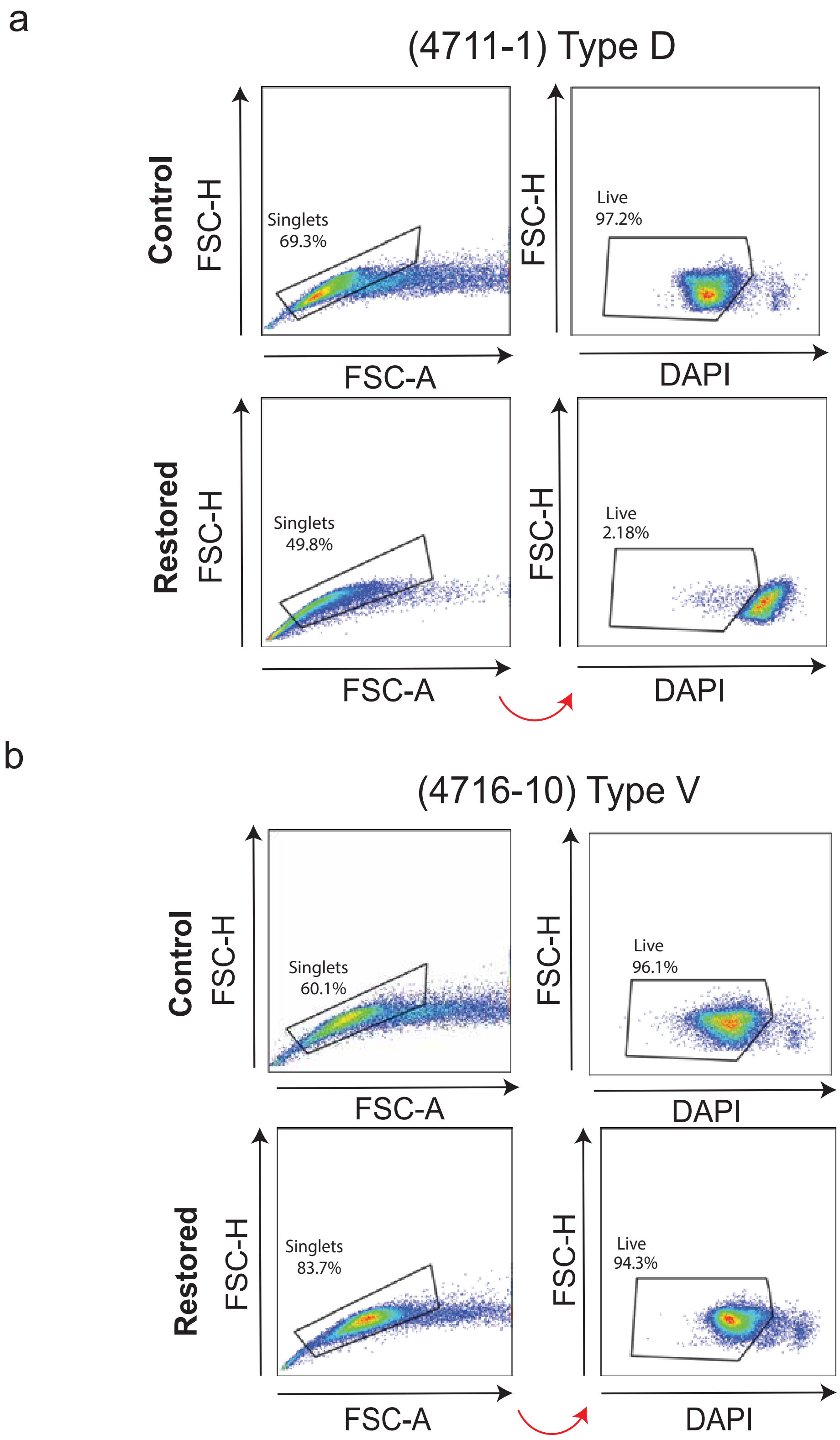

Supplementary Fig. 3. Gating scheme for cell viability assays. (**A,B**) Gating strategy for determining cell viability after p53 reactivation in Type D (4711-1) and Type V (4716-10) cells. Doublets are excluded based on FSC properties. DAPI-negative cells indicate percentage of live cells.

Supplementary Figure 4

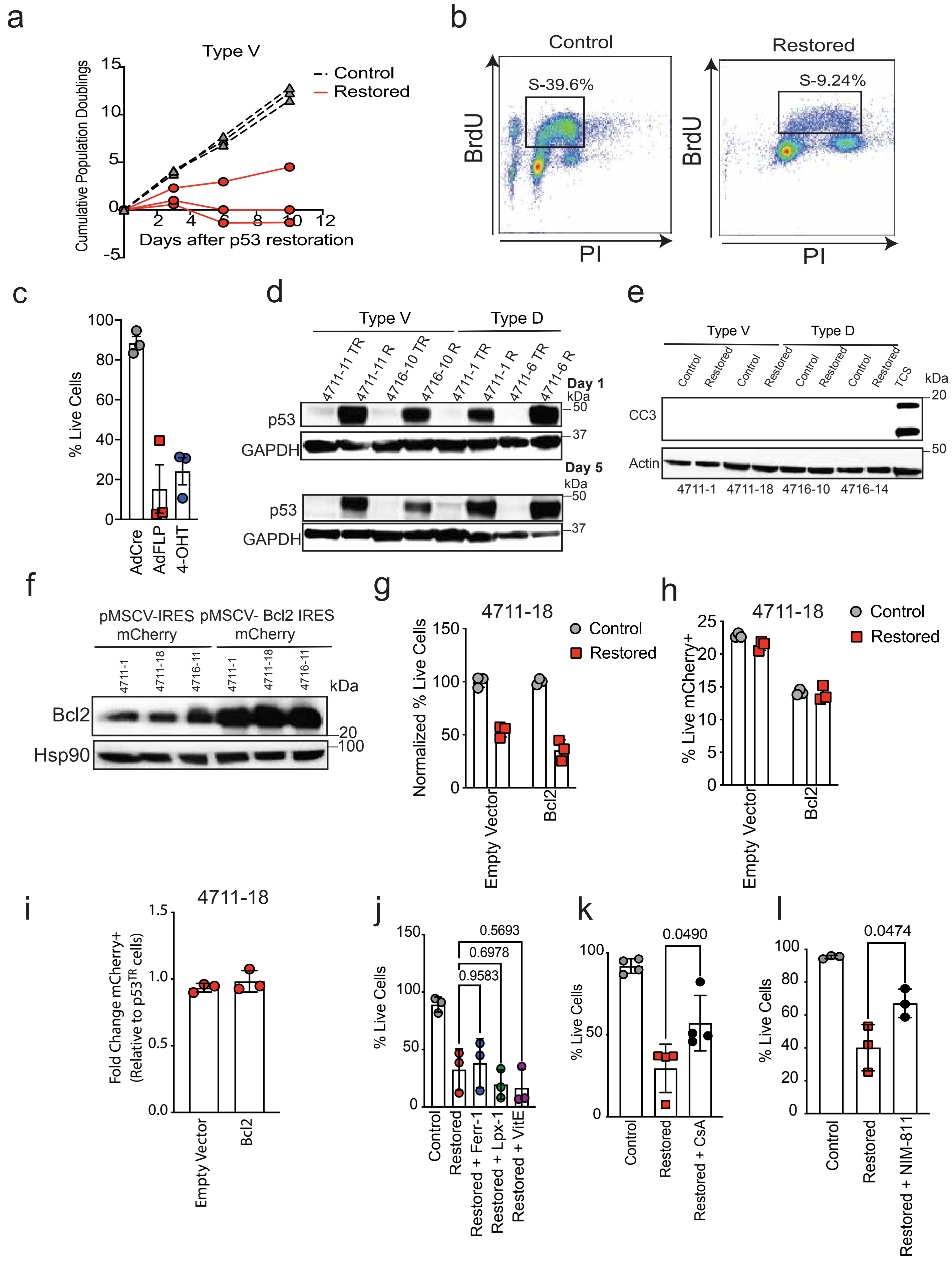

Supplementary Fig. 4. p53-reactivation induces cyclophilin-mediated death or senescence in tumor-derived SCLC cell lines. (**A**) Cumulative population doublings of Type V (4711-11, 4716-10, 4716-14) cells in **Fig.3F**. 150,000 cells plated for n=3 Type V cell lines at t=0 hrs. 150,000 cells cells were re-plated at t=3 days and t=6 days. Each symbol represents the mean of n=3 technical replicates per Type V cell line. (**B**) Flow cytometry plots of BrdU incorporation in Type V cells in **Fig.3G**. Percentage of cells in S-phase (BrdU+) was calculated in Type V cells (n=3) 72hrs after 4-OHT treatment. (**C**) Flow cytometry-assisted cell viability assay in Type D cells 4 days after 4-OHT treatment, or Ad:FlpO treatment. Ad:CMV-Cre treatment used as a negative control for p53 reactivation. Each symbol represents the mean of n=3 technical replicates from a Type D cell line. Distinct Type D cell lines (4711-1, 4711-6, 4716-11) were used as biological replicates. Error bars represent mean ± SEM. (**D**) Immunoblot analysis for expression of p53 in Type V (4711-11, 4716-10) and Type D (4711-1, 4711-6) cell lines one or five days after 4-OHT treatment. n=2 representative cell lines used per cell line. GAPDH used as loading control. (**E**) Immunoblot analysis for apoptosis markers in Type V (4716-10, 4716-14) and Type D (4711-1, 4711-18) cells 24 hours after 4-OHT treatment. Positive control (TCS) treated with TNF-α (1µg/mL), Smac mimetic (SM-164; 100nM), and cyclohexamide (10µg/mL) for 8 hours. Actin is loading control. **(F)** Immunoblot analysis for Bcl2 from Type D (4711-1, 4711-18, 4716-11) cells transduced with pMSCV-IRES mCherry or pMSCV-Bcl2 IRES mCherry. Hsp90 is loading control. (**G**) Flow cytometry-assisted cell viability assay in representative Type D (4711-18) cell line overexpressing Bcl2 after 3 days of 4-OHT treatment. Live cell percentage determined by quantification of DAPI negative population; n=3 replicates. Error bars represent mean ± s.d. Experiment was conducted in n=2 independent Type D cell lines.(**H,I**) Percentage (**h**) and fold change (**i**) in the proportion of mCherry-positive live cells shown in **(g). J**) Type D cells were treated with 4-OHT and ferroptosis inhibitors for 4 days. Percentage of live cells was determined using flow cytometry by quantification of DAPI negative population. Each symbol represents the mean of n=3 technical replicates from a Type D cell line. Distinct Type D cell lines (4711-6, 4711-18, 4716-11) were used as biological replicates. Statistical significance was determined by one-way ANOVA followed by Dunnett's multiple comparison test. Error bars represent mean ± s.d (**K**) Type D cells were treated with 4-OHT and CsA for 3-5 days. Percentage of live cells was determined using flow cytometry by quantification of DAPI negative population. Each symbol represents the mean of n=3 technical replicates from a Type D cell line. Distinct Type D cell lines (4711-1, 4711-6, 4711-18, 4716-11) were used as biological replicates. Statistical significance was determined by two-tailed Student's *t*-test. Error bars represent mean ± s.d. (**L**) Type D cells were treated with 4-OHT and NIM-811 for 3 days. Percentage of live cells was determined using flow cytometry by quantification of DAPI negative population. Each symbol represents the mean of n=3 technical replicates from a Type D cell line. Distinct Type D cell lines (4711-1, 4711-18, 4716-11) were used as biological replicates. Statistical significance was determined by two-tailed Student's *t*-test. Error bars represent mean ± s.d. Source data are provided as a Source Data file.

Supplementary Figure 5

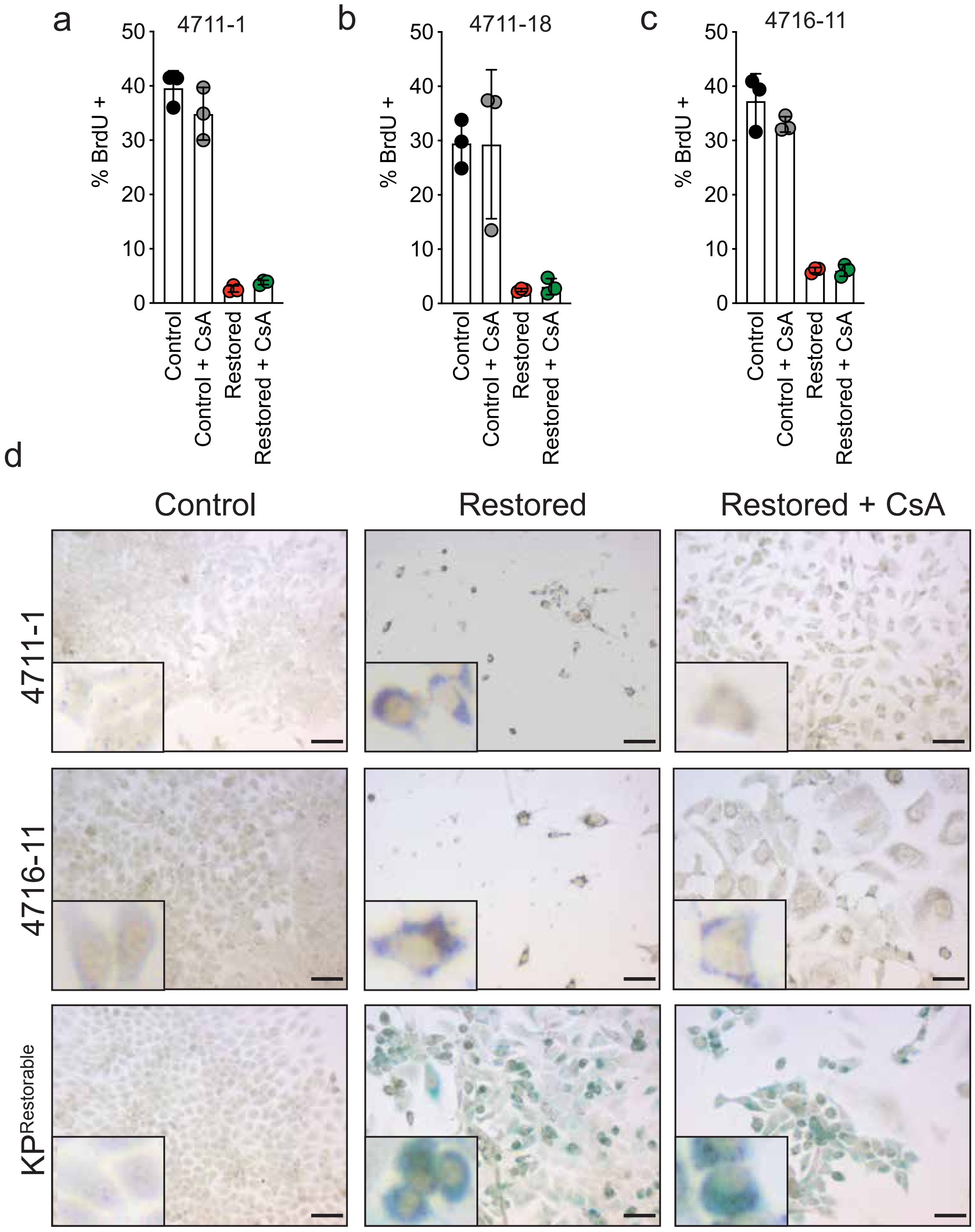

Supplementary Fig. 5. Cyclophilin inhibition does not promote cell proliferation in Type D SCLC. (**A-C**) Quantification of BrdU+ cells 72 hours after 4-OHT and CsA treatment in Type D cell lines (4711-1, 4711-18, 4716-11). Each symbol represents a technical replicate (n=3). Error bars represent mean ± s.d. (**D**) Brightfield photomicrographs of SA-β-Gal stained Type D (4711-1, 4716-11) cells after 4-OHT and CsA treatment. $KP^{Restorable}$ cell line used as a positive control for SA-β-Gal staining. Scale bars, 25μm; insets are magnified 5×. Source data are provided as a Source Data file.

Supplementary Figure 6

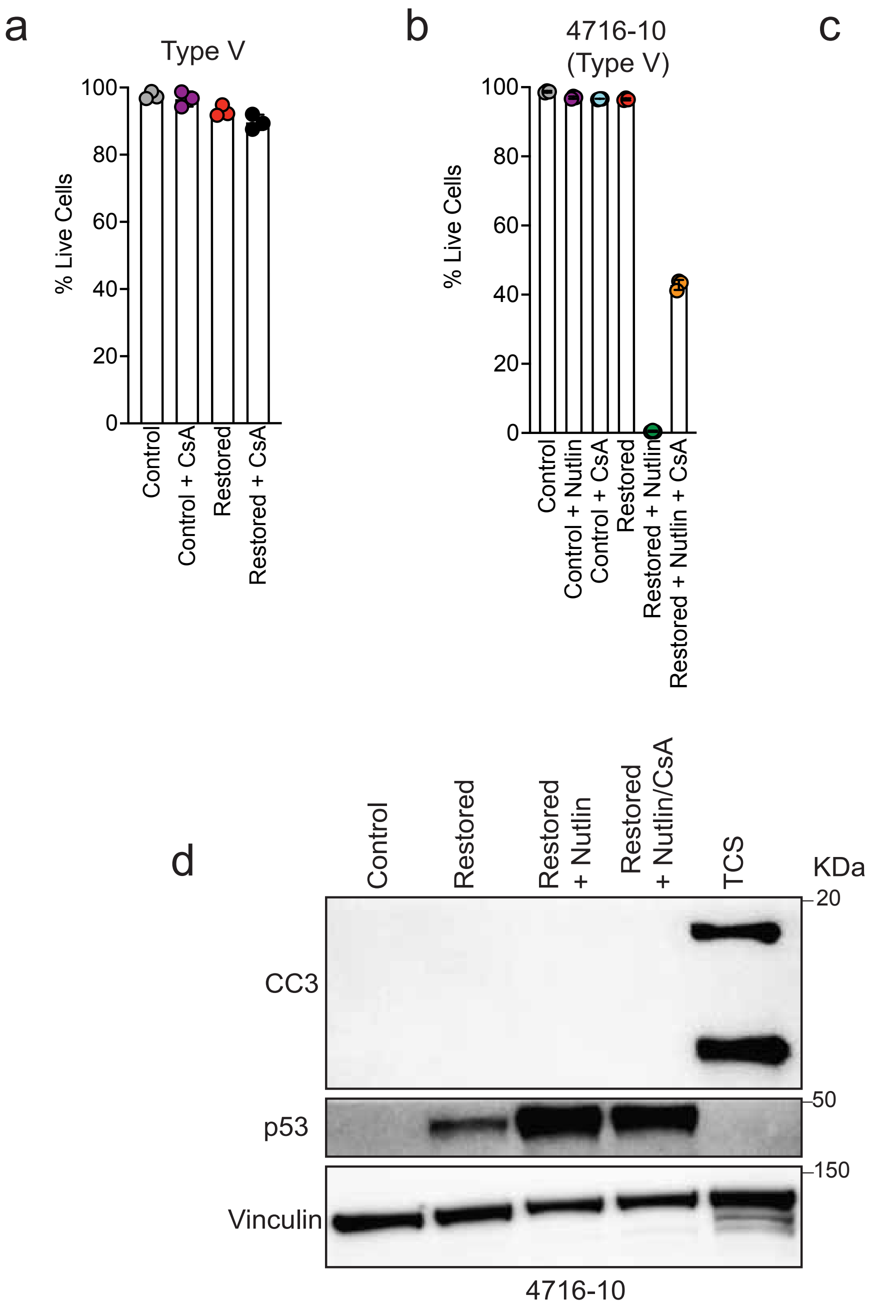

Supplementary Fig. 6. Nutlin-3a sensitizes SCLC cells to p53-mediated death. (**A**)Type V (4711-11, 4716-10, 4716-14) cells were treated with 4-OHT and CsA for 3 days. Percentage of live cells was determined using flow cytometry by quantification of DAPI negative population. Each symbol represents a biological replicate of n=3 Type V cell lines. Error bars represent mean ± s.d. (**B,C**) Representative Type V (4716-10) and Type D (4711-18) cells were treated with 4-OHT, CsA, and/or Nutlin-3a for 3 days. Percentage of live cells was determined using flow cytometry by quantification of DAPI negative population; n=3 technical replicates. Error bars represent mean ± s.d. Experiment was conducted twice per SCLC subtype. (**D**) Immunoblot analysis for apoptotic marker and p53 in representative SCLC cell line treated with 4-OHT, Nutlin-3a, and/or CsA for 48hrs. Vinculin is loading control. Source data are provided as a Source Data file.

Supplementary Figure 7

a

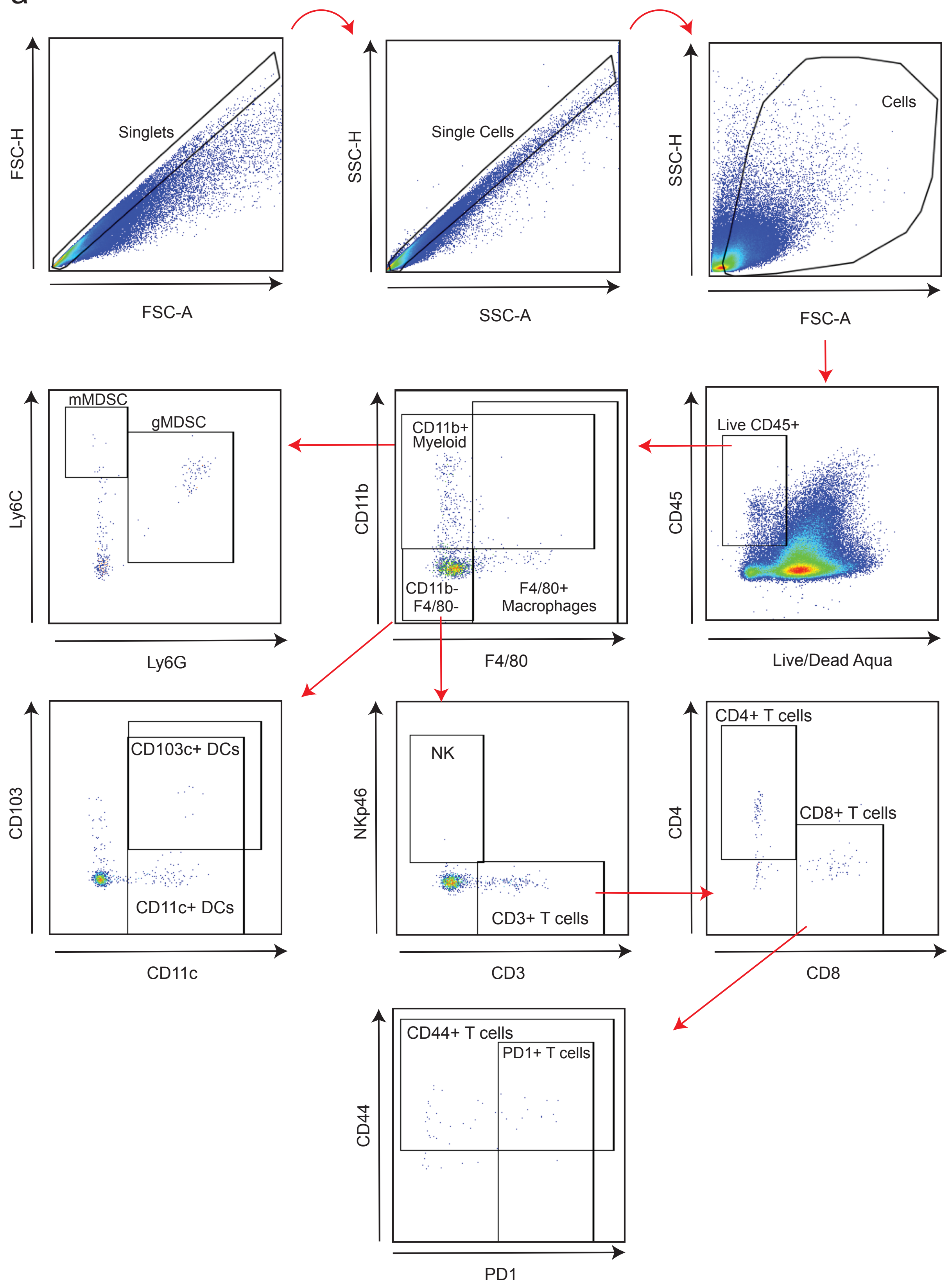

Supplementary Fig. 7. Gating scheme for *in vivo* immune infiltration. (A) Gating strategy for quantifying immune cell infiltration 72 hrs after p53 reactivation in *RP*^{TR}*R2* mice. Doublets and debris are excluded based on FSC and SSC properties. Live immune cells are identified based on CD45+ and Live/ Dead Aqua -. Immune cell populations are classified as follows: F4/80+ Macrophages (CD45+F480+), CD11b+ Myeloid cells (CD45+CD11b+), granulocytic Myeloid-derived suppressor cells (gMDSC: CD45+CD11b+ Ly6G+Ly6C+), monocytic Myeloid-derived suppressor cells (mMDSC: CD45+CD11b+ Ly6G-Ly6C+), CD11c+ Dendritic Cells (DCs: CD45+CD11b-F480-CD11c+), CD103+ DCs (CD45+CD11b-F480-CD11c+CD103+), Natural Killer cells (NK: CD45+CD11b-F480-CD3-NKp46+), CD3 T cells (CD45+CD11b-F480-CD3+NKp46-), CD4 T cells (CD45+CD11b-F480-NKp46-CD3+CD4+), CD8 T cells (CD45+CD11b-F480-NKp46-CD3+CD8+), CD44+ CD8 T cells (CD45+CD11b-F480-NKp46-CD3+CD8+CD44+), PD1+ CD8 T cells (CD45+CD11b-F480-NKp46-CD3+CD8+PD1+).

Supplementary Figure 8

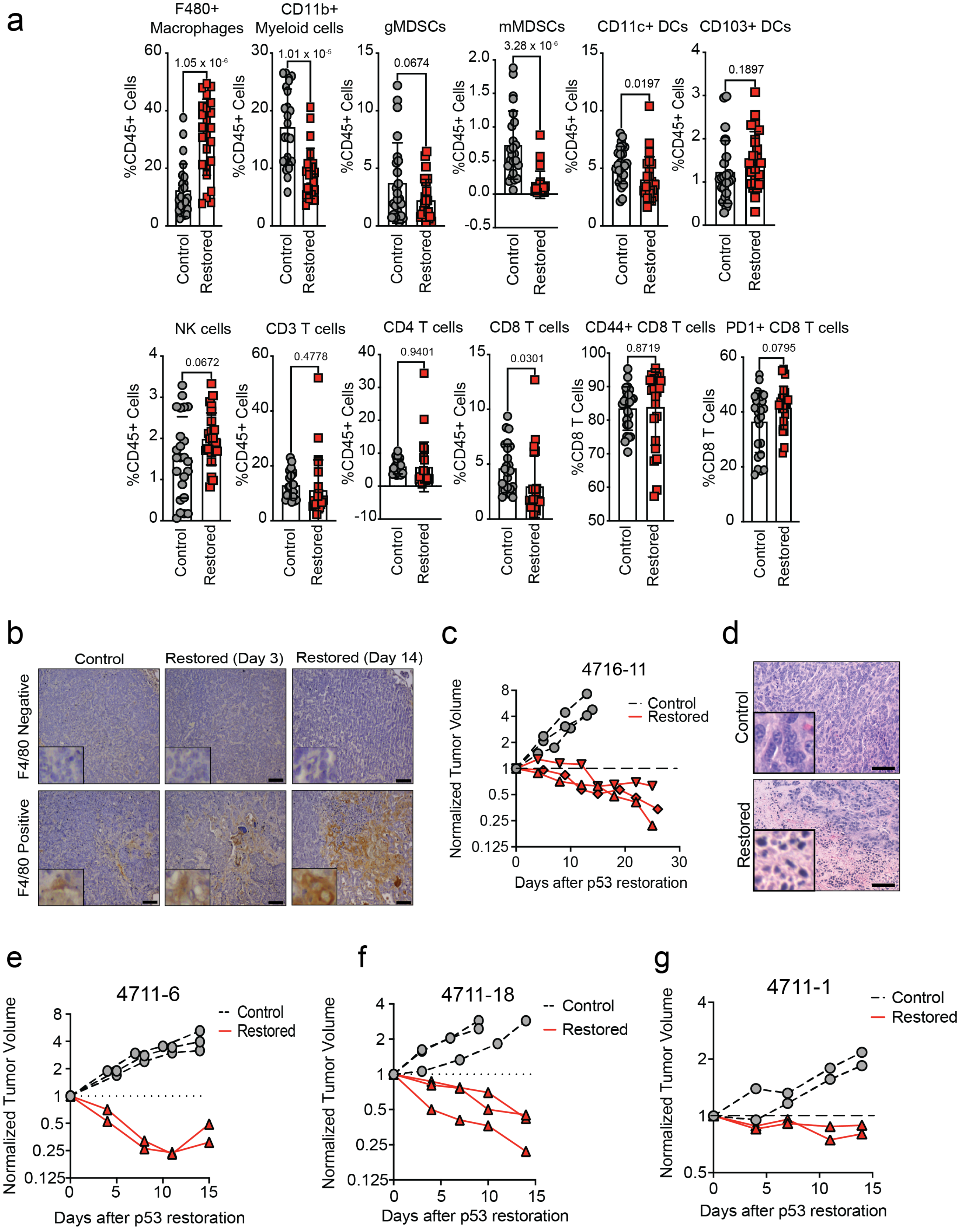

Supplementary Fig. 8. p53 reactivation per se does not induce widespread immune inflammation to mediate Type D cell death.(**A**) Flow cytometry analysis of macrophage, myeloid-derived suppressor cells (MDSCs), dendritic cells (DC), natural killer cells (NK), and T-cell infiltration in *RP^TR^R2* tumors 3 days after vehicle (Control) or tamoxifen (Restored) treatment. n=24 tumors from 4 Control mice, n= 24 tumors from 3 Restored mice. Statistical significance was determined by two-tailed Student's *t*-test. Error bars represent mean ± s.d. (**B**) Representative photomicrographs for F4/80 IHC in Control or Restored RP^X^R2 tumors. Scale bars, 25μm: insets are magnified 5× (**C**) Representative growth curve from Type D (4716-11) allograft tumor(s). Each line represents allograft tumors (n=3) of one Type D cell line. (**D**) Brightfield photomicrographs of allograft tumors generated from Type D cell line used in (**c**) 7 days after tamoxifen treatment. Each condition had n=2 tumors. Scale bars, 25μm. (**E-G**) Growth curve analyses from additional Type D (4711-6, 4711-18, 4711-1) allograft tumors. Experiment was conducted in n=3 Type D cell lines with at least n=2 tumors per treatment group. Source data are provided as a Source Data file.

Supplementary Figure 9

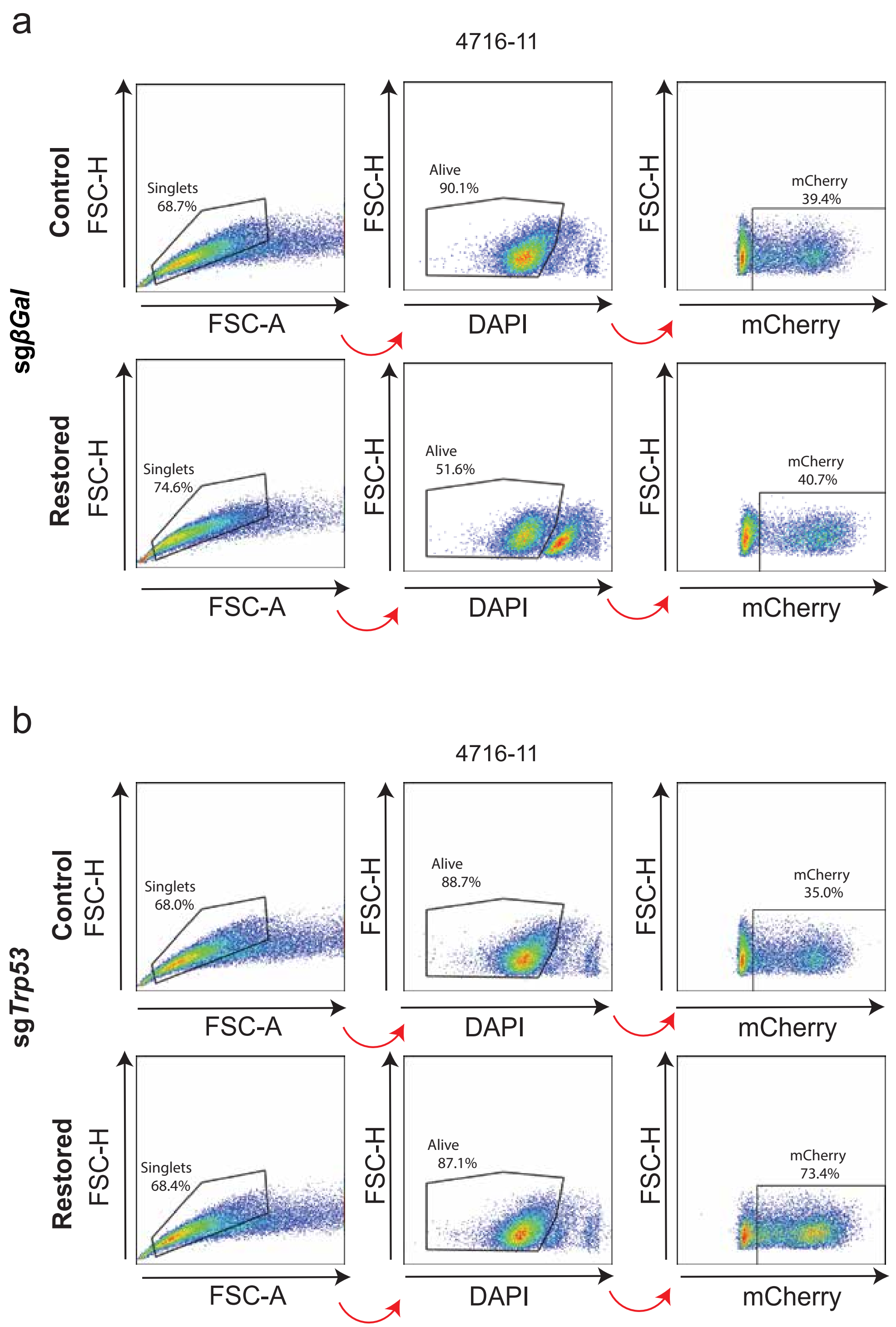

Supplementary Fig. 9. Gating scheme for LentiCRISPRv2-mCherry CRISPR Screen. (**A,B**) Gating strategy for enrichment for mCherry positive cells after p53 reactivation in Type D (4716-11) cells transduced with LentiCRISPRv2-mCherry constructs targeting βGal (**a**) and sgp53 (**b**). Doublets are excluded based on FSC properties. Dead cells are excluded based on DAPI staining. Percentage of mCherry positive cells from DAPI-negative population is calculated in control and restored samples to determine fold change in the proportion of mCherry-positive cells.

Supplementary Figure 10

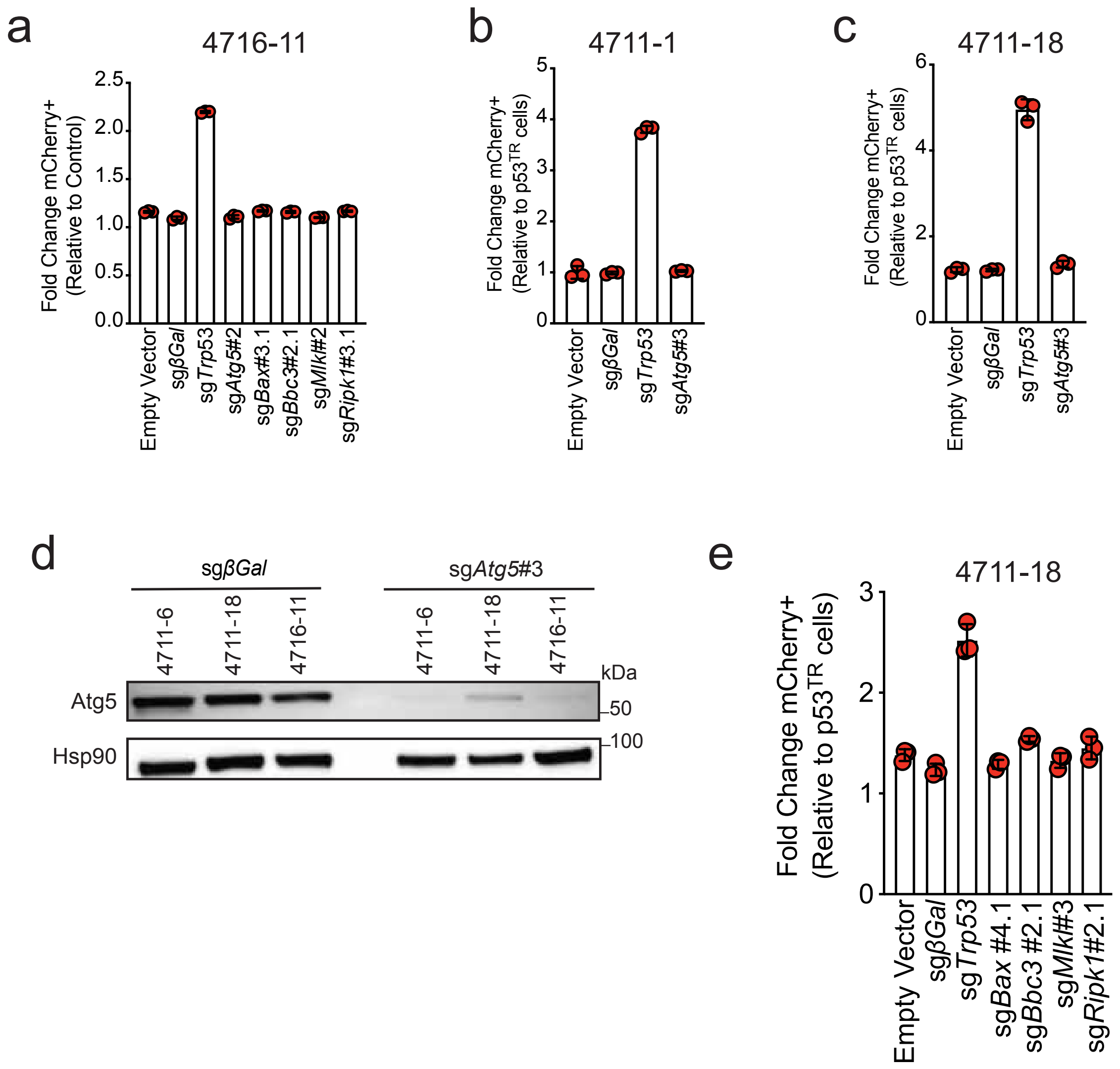

Supplementary Fig. 10. CRISPR-mediated knockout of critical regulators of autophagy, apoptosis, and RIPK1-mediated necroptosis does not block p53-mediated death in Type D cells. (**A**) Fold change in the proportion of mCherry-positive cells 72hrs after 4-OHT treatment in a Type D (4716-11) cell line expressing sgRNAs targeting distinct autophagy (*Atg5*), apoptosis (*Bax, Bbc3*) and necroptosis (*Mlkl, Ripk1*) regulators. Empty Vector, β-Gal, and a p53 targeting sgRNA used as controls. Each symbol represents a technical replicate (n=3). Error bars represent mean ± s.d. (**B,C**) Fold change in the proportion of mCherry-positive cells 4 days after 4-OHT treatment in two additional Type D (4711-1, 4711-18) cell lines expressing an sgRNA targeting Atg5. Empty Vector, β-Gal, and a p53 targeting sgRNAs used as controls. Each symbol represents a technical replicate (n=3). Error bars represent mean ± s.d. (**D**) Immunoblot analysis for Atg5 in three distinct Type D (4711-6, 4711-18, 4716-11) cell lines expressing β-Gal or an *Atg5* targeting sgRNA. Hsp90 is loading control. (**E**) Fold change in the proportion of mCherry-positive cells 4 days after 4-OHT treatment in additional Type D (4711-18) cell line expressing sgRNAs targeting *Bax*, *Bbc3, Mlkl,* and *Ripk1*. Empty Vector, β-Gal, and a p53 targeting sgRNAs used as controls. Each symbol represents a technical replicate (n=3). Error bars represent mean ± s.d. Source data are provided as a Source Data file.

Supplementary Figure 11

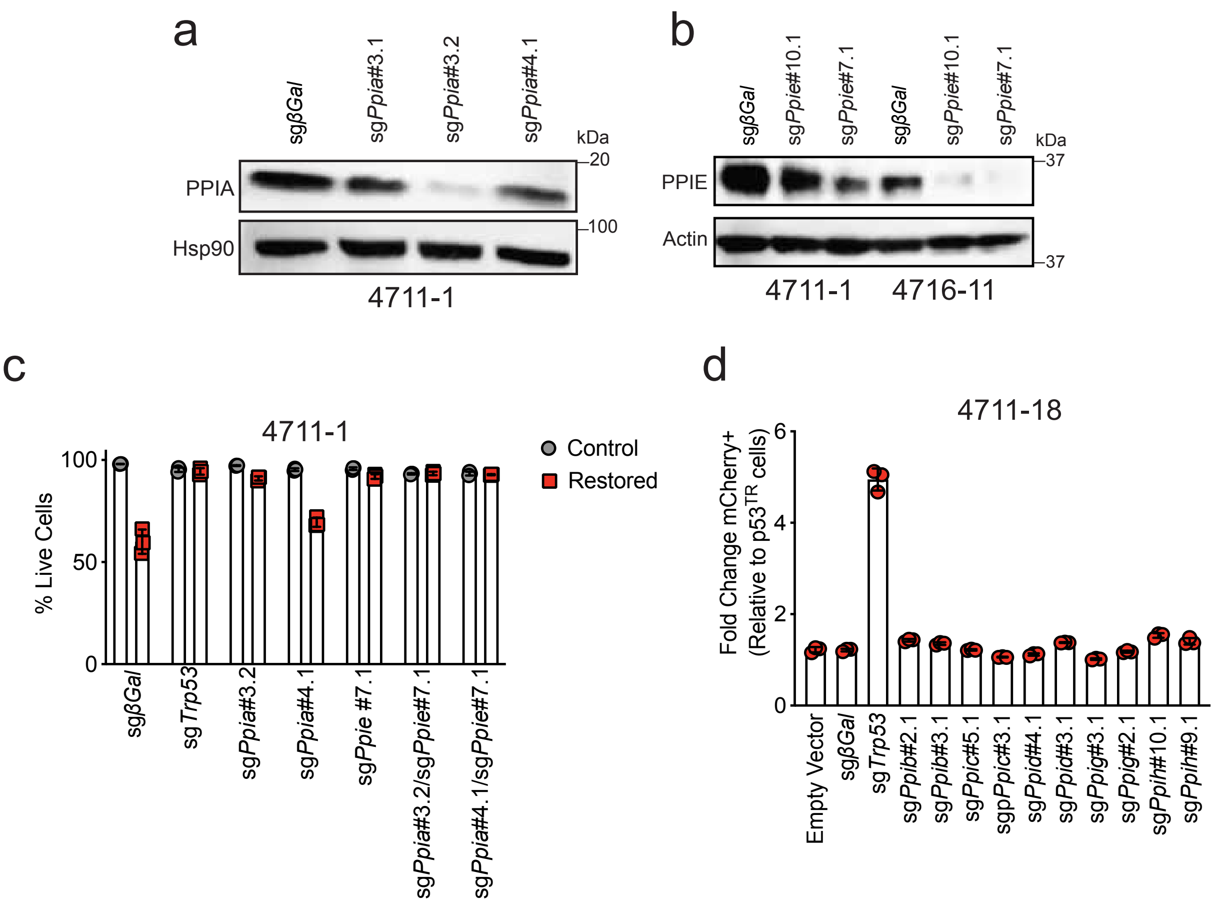

Supplementary Fig. 11. Cyclophilins A and E are effectors of p53-mediated Type D cell death. (**A**,**B**) Immunoblot analysis for cyclophilin A (**a**) and cyclophilin e (**b**) in Type D (4711-1,4716-11) cell lines expressing sgRNAs targeting β-Gal, *Ppia*, or *Ppie*. Hsp90 and actin are loading controls. (**C**) Flow cytometry-assisted cell viability assay in Type D (4711-1) cell line expressing sgRNAs targeting cyclophilin A, cyclophilin E, or both, 4 days after 4-OHT treatment. Live cell percentage determined by quantification of DAPI negative population. β-Gal, and a p53 targeting sgRNA used as controls. Each symbol represents a technical replicate (n=3). Error bars represent mean ± s.d. (**D**) Fold change in the proportion of mCherry-positive cells 4 days after 4-OHT treatment in additional Type D (4711-18) cell line expressing sgRNAs targeting distinct cyclophilins. Empty Vector, β-Gal, and a p53 targeting sgRNA used as controls. Each symbol represents a technical replicate (n=3). Error bars represent mean ± s.d. Source data are provided as a Source Data file.

Supplementary Figure 12

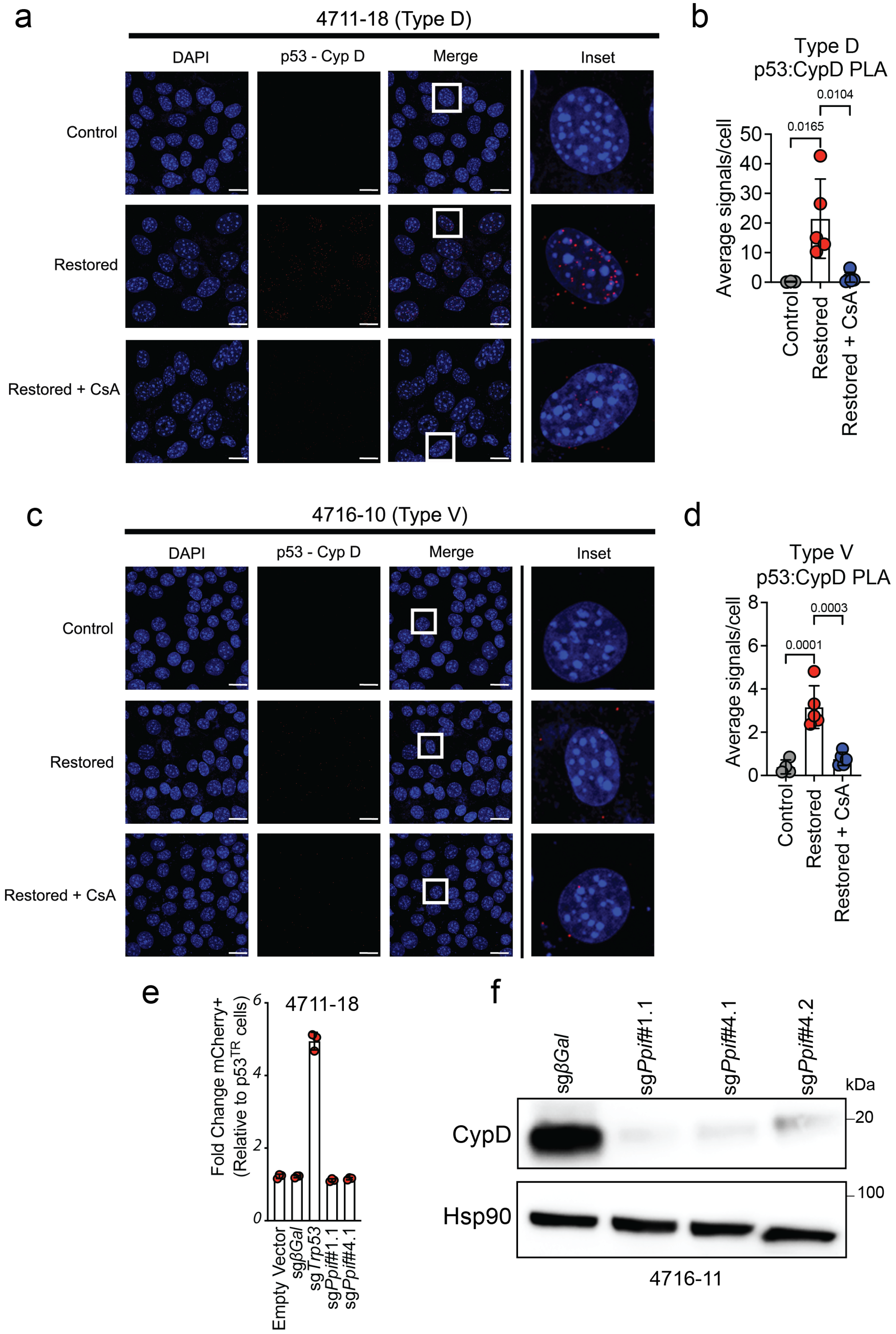

Supplementary Fig. 12. Cyclophilin D is not required for p53-mediated death in Type D SCLC. (**A-D**) Type D (4711-18) (**a**) and Type V (4716-10) cells (**c**) were treated with 4-OHT and CsA for 48 hours and p53-Cyclophilin D (CypD) interactions were assessed using proximity ligation assay. Representative images are shown for each condition (scale bar, 30μm). White boxes denote the enlarged insets. Quantification of the average number of PLA signals per cell shown in (**b,d**).Values are expressed as mean ± SD. *n* = 3-5 random fields of view (>100 cells). Statistical significance was determined by one-way ANOVA followed by Tukey's multiple comparisons test.(**E**) Fold change in the proportion of mCherry-positive cells 4 days after 4-OHT treatment in a representative Type D (4711-18) cell line expressing sgRNAs targeting exons 1 and 4 of the *Ppif* gene. Empty Vector, β-Gal, and a p53 targeting sgRNA used as controls. Each symbol represents a technical replicate (n=3). Error bars represent mean ± s.d. Conducted in n=2 independent Type D cell lines. (**F**) **I**mmunoblot analysis for cyclophilin D (CypD) in a Type D (4716-11) cell line expressing β-Gal or three distinct *Ppif* targeting sgRNAs. Hsp90 is loading control. Source data are provided as a Source Data file.

Supplementary Figure 13

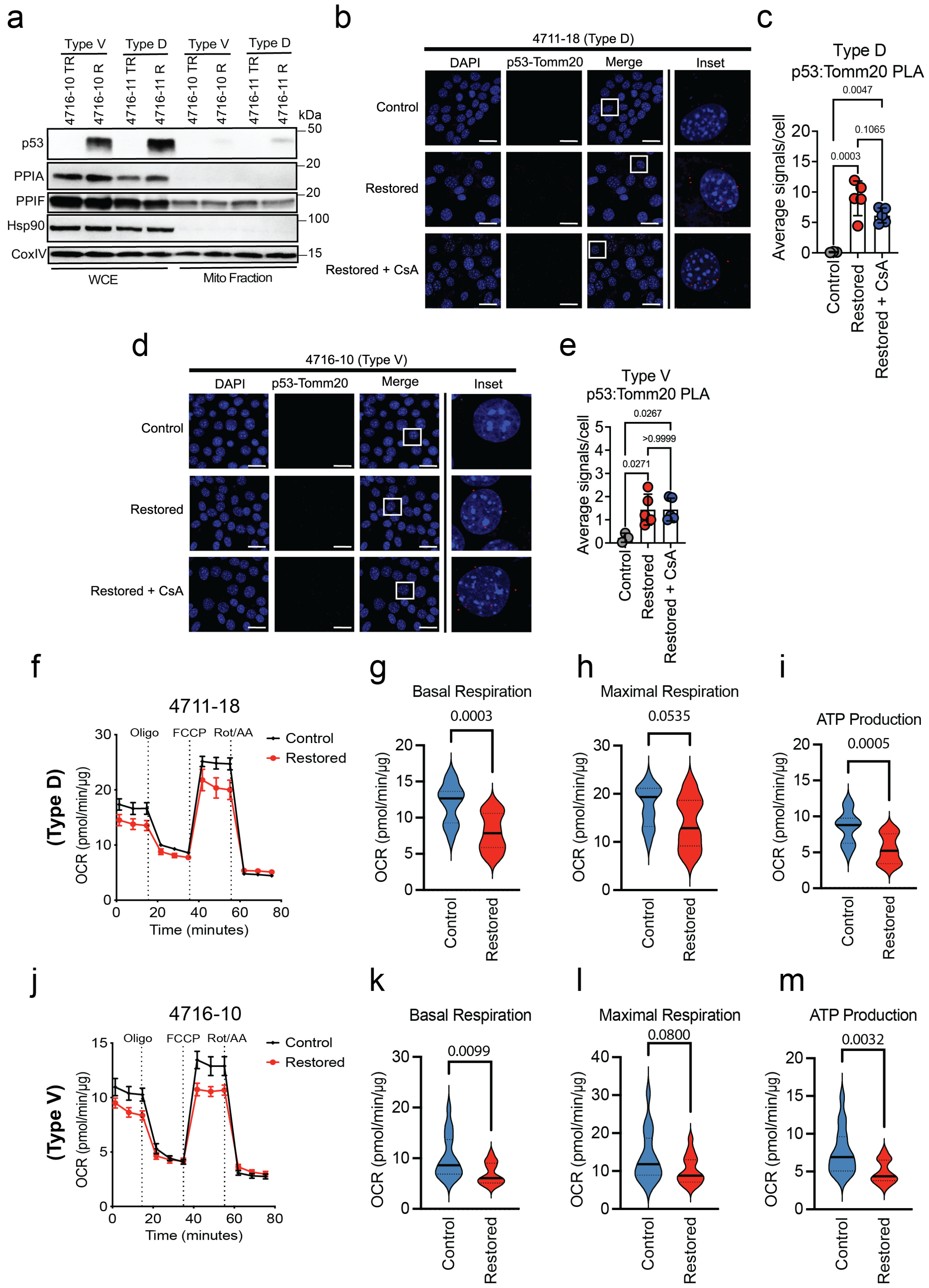

Supplementary Fig. 13. p53 does not prominently associate with the mitochondria and mitochondria are not dysfunctional during Type D SCLC cell death. (**A**) Immunoblot analysis from a Type D (4716-11) or Type V (4716-10) cell line for PPIA, PPIF, and p53 expression in whole cell extracts (WCE) and mitochondrial fractions after 4 days of 4-OHT treatment. Hsp90 used as loading control for WCE; COXIV used as a loading control for mitochondrial fraction. (**B-E**) Type D (4711-18) (**b**) and Type V (4716-10) cells (**d**) were treated with 4-OHT and CsA for 48 hours and p53-Tomm20 interactions were assessed using proximity ligation assay. Representative images are shown for each condition (scale bar, 30μm). White boxes denote the enlarged insets. Quantification of the average number of PLA signals per cell shown in (**c,e**).Values are expressed as mean ± SD. *n* = 3-5 random fields of view (>100 cells). Statistical significance was determined by one-way ANOVA followed by Tukey's multiple comparisons test. Seahorse XF cell mitochondrial stress test assay performed in Type D (**F**) and Type V (**J**) cells after treatment with vehicle or 4-OHT. (**F,J**) OCR profile plots. (**G,K**) Basal respiration. (**H,L**) Maximal respiration. (**I,M**) ATP production. Relative oxygen consumption rate was normalized to protein abundance. Each symbol in OCR profile plots represents the mean of at least n=4 technical replicates of three reading cycles. Statistical significance was determined by two-tailed Student's *t*-test. Error bars represent s.d. Experiment was conducted in n=3 cell lines per subtype (Type D: 4711-6, 4711-18, 4716-11. Type V: 4711-7, 4716-10, 4716-14). Source data are provided as a Source Data file.

Supplementary Figure 14

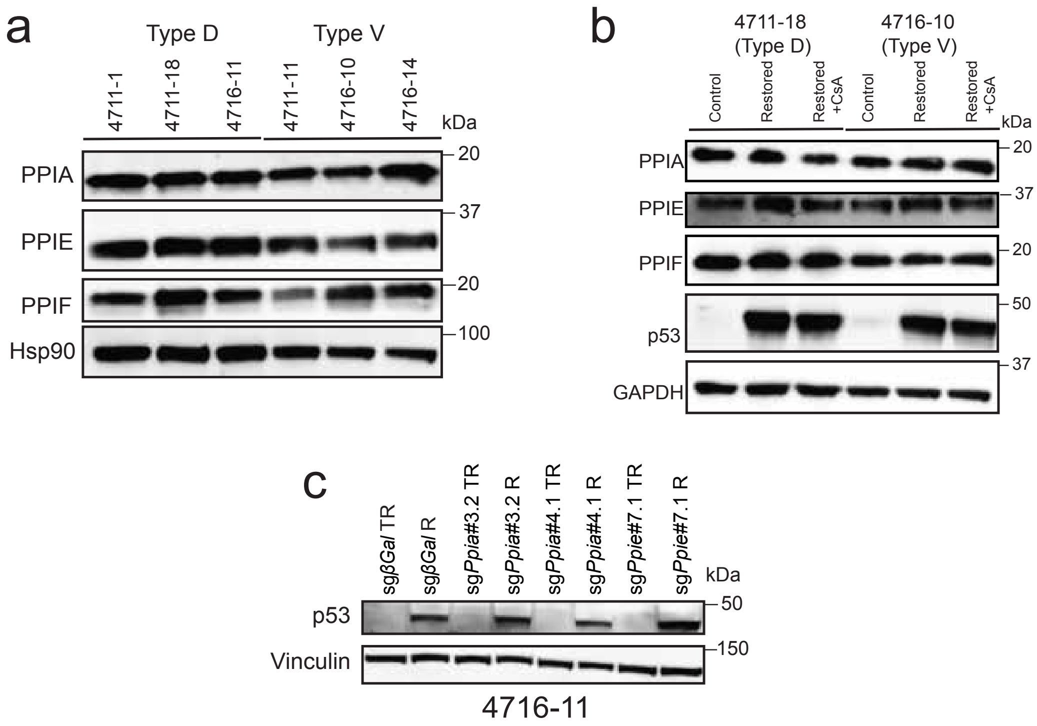

Supplementary Fig. 14.Cyclophilins are similarly expressed across SCLC subtypes and do not prominently alter p53 stability. (**A**) Immunoblot analysis for distinct cyclophilins in three distinct Type D (4711-1,4711-18, 4716-11) or Type V cell lines. Hsp90 is loading control. (**B)** Immunoblot analysis for distinct cyclophilins and p53 in Type D (4711-18) or Type V (4716-10) cell lines 24hrs after 4-OHT and CsA treatment. GAPDH is loading control. (**C**) Immunoblot analysis for p53 in Type D (4716-11) cell line expressing sgRNAs targeting β-Gal, *Ppia*, or *Ppie* 72 hours after 4-OHT treatment. Source data are provided as a Source Data file.

Supplementary Figure 15

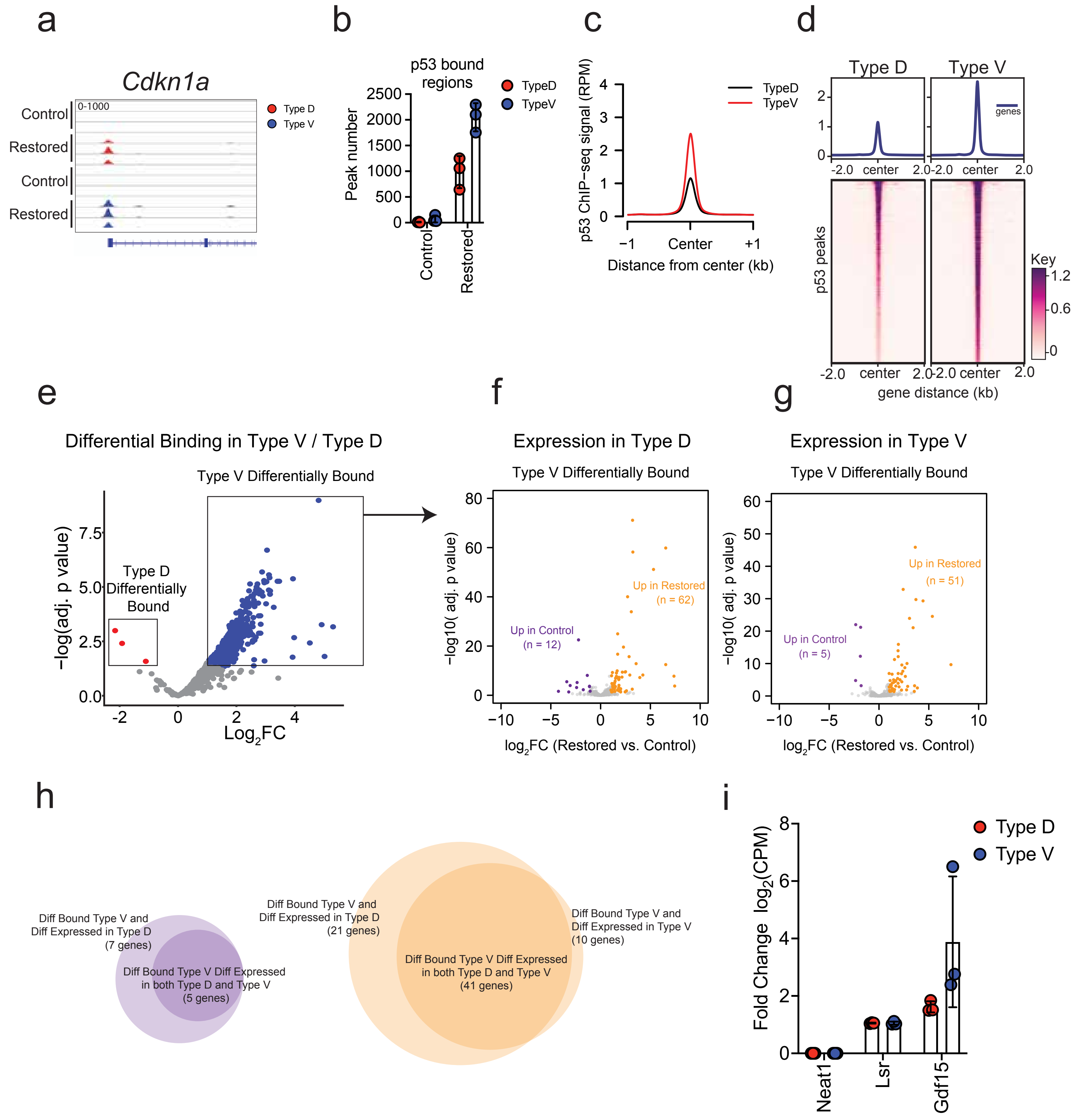

Supplementary Fig. 15. p53 binding does not identify subtype-specific transcriptional programs. (**A**) Genome browser view of p53 ChIP-seq signal at a canonical p53-target gene, *Cdkn1a.* (**B**) Quantification of p53 bound regions in Control and Restored cells. n=3 for both Type D (4711-1, 4711-18, 4716-11) and Type V (4711-11, 4716-10, 4716-14) cells. (**C**) Comparison of p53 ChIP-seq signal between Type D and Type V cells. Data are centered on p53 bound peaks across a ±1kb window. (**D**) Heatmap representation of p53 bound chromatin peaks 48hrs after 4-OHT treatment in Type D (n=3) and Type V cells (n=3). Heatmaps are centered on p53-bound peaks across a ±2kb window. (**E**) Volcano plot of differentially p53 bound regions in Type V (n=1032 genomic regions; blue) and Type D (n=3 genomic regions; red) cells. (**F,G**) Volcano plots of RNA-sequencing gene expression from Type D (**f**) and Type V (**g**) cells of Type V differentially bound p53 regions identified in (**e**). Colored dots represent genes that are differentially enriched ($\log_2$ fold-change greater-than 1 and false discovery rate (FDR)-adjusted *P*-value less-than 0.05) in Restored (orange) or Control (purple). (**H**) Venn diagrams comparing differentially bound Type V genes and their differential expression in Control (purple) or Restored (orange) cells. **(I**) Quantification of $\log_2$ fold change in counts per million (CPM) of Type D differentially bound genes identified in (**f**) in Type D (n=3) and Type V (n=3) cells. Error bars represent mean ± s.d. Source data are provided as a Source Data file.

Supplementary Figure 16

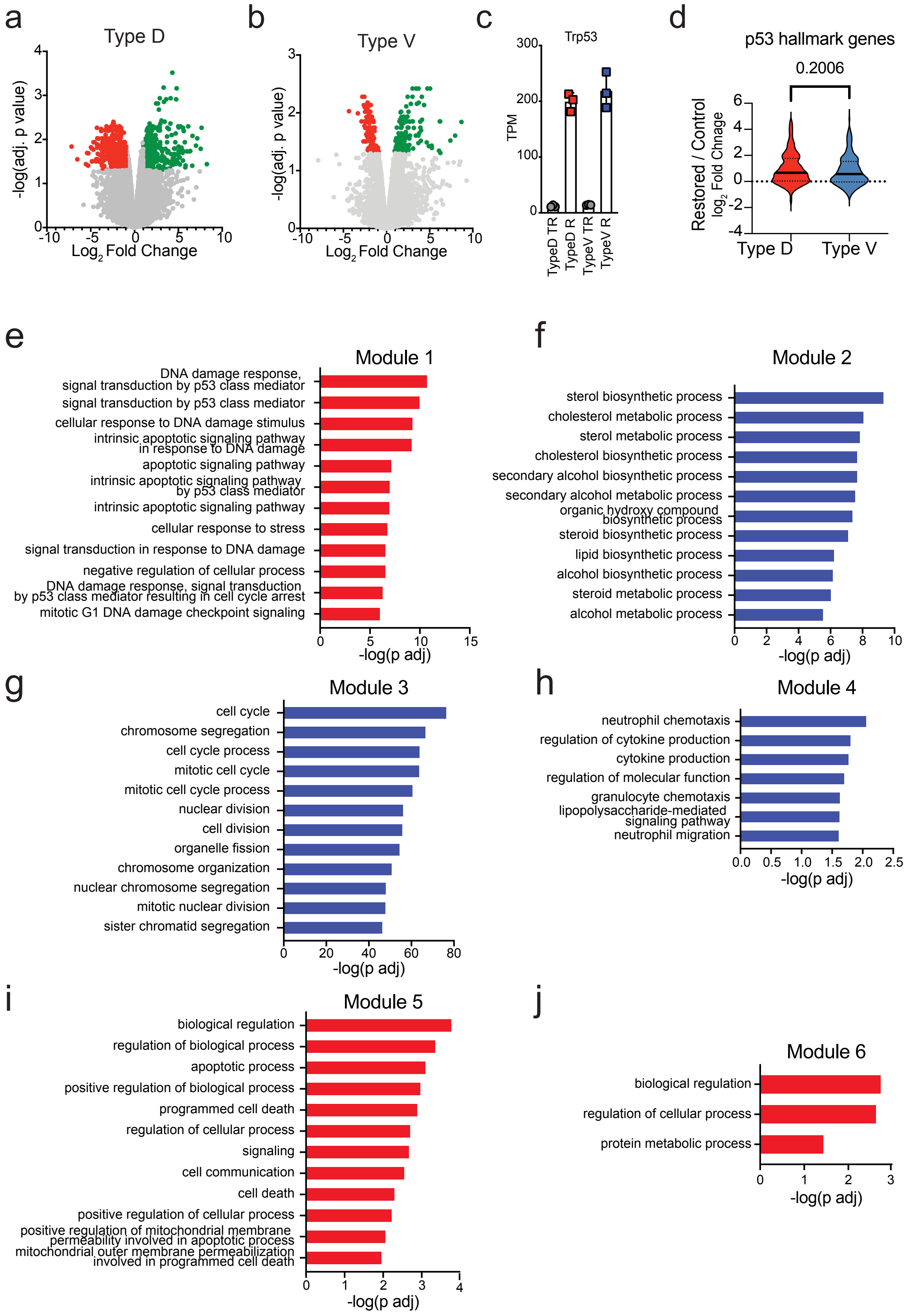

Supplementary Fig. 16. p53 reactivation modulates diverse biological processes in SCLC. (**A,B**) Volcano plots of RNA-sequencing data in Type D (**a**) and Type V (**b**) cells. Colored dots represent genes that are differentially enriched (log_2 fold-change greater than 1 and false discovery rate (FDR)-adjusted *P*-value less-than 0.05) in Restored (n=401, green) or Control (n=363, red) cells. n=3 for both Type D (4711-1, 4711-18, 4716-11) and Type V (4711-11, 4716-10, 4716-14) cells. (**C**) Quantification of transcripts per million (TPM) for *Trp53* in Control and Restored Type D (n=3) and Type V (n=3) cell lines. Error bars represent mean ± s.d.(**D**) Violin plots indicating log_2 fold-change in expression of canonical p53 targets from the GSEA gene set 'HALLMARK_P53_PATHWAY' in Restored Type D (n=3) and Type V (n=3) cells. Statistical significance was determined by two-tailed Student's *t*-test.(**E-J**) Bar plots indicating -log(adj.*p* value) of top GO: Biological Processes signatures associated with gene expression modules 1-6 identified in (Figure 6A). Gene ontology analysis was conducted using the g:Profiler web tool. Adjusted p-value was calculated using Fisher's one-tailed test by g:Profiler web tool. Red bar graphs represent genes enriched in Restored cells after p53 reactivation, while blue bar graphs represent genes enriched in Control cells. Source data are provided as a Source Data file.

Supplementary Figure 17

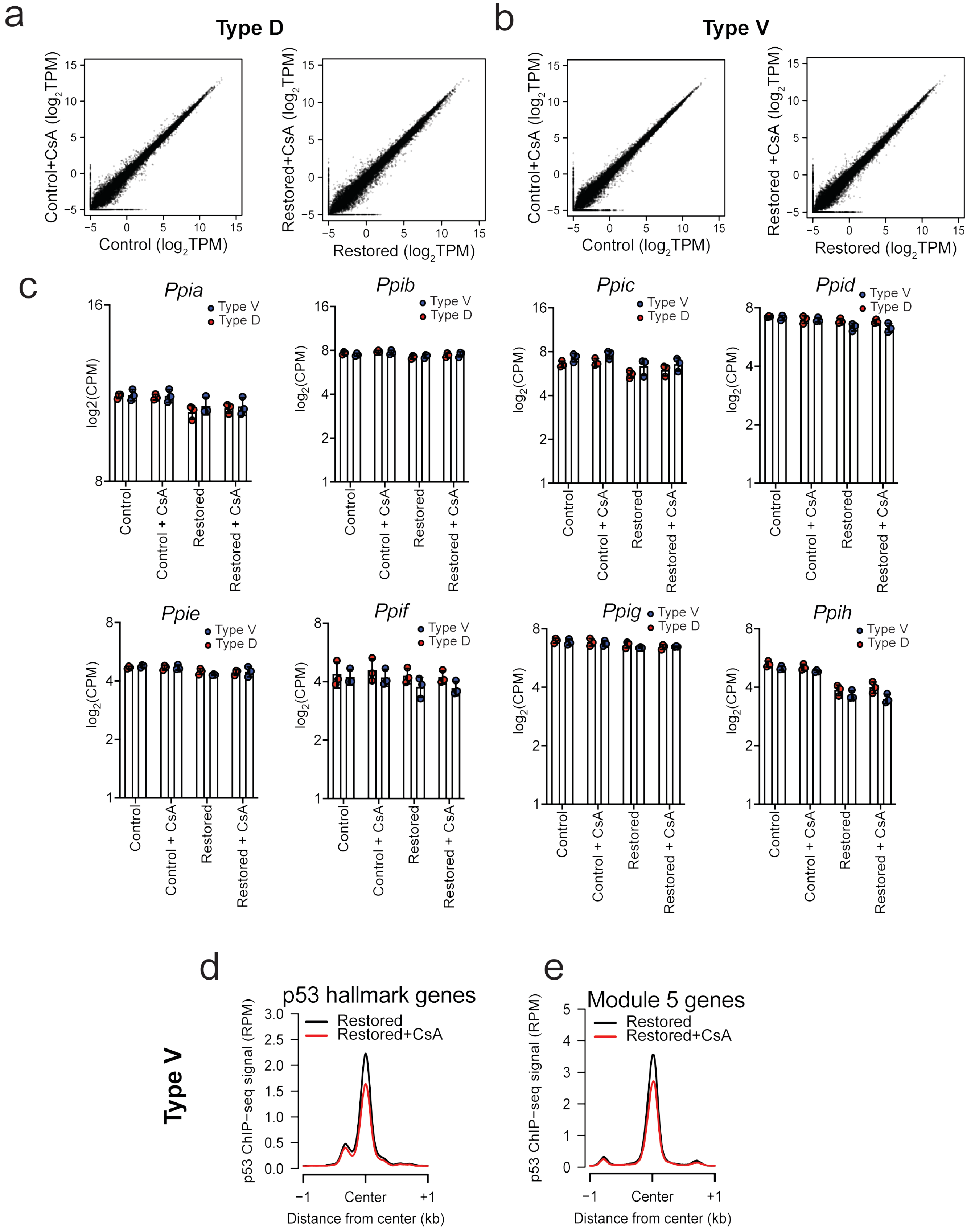

Supplementary Fig. 17. CsA does not influence individual gene transcription or p53 binding. (**A,B**) Dot plot analysis of gene expression 72hrs after CsA treatment in Control and Restored Type D (4711-1, 4711-18, 4716-11) (**a**) and Type V (4711-11, 4716-10, 4716-14) (**b**) cells. Mean expression from n=3 cell lines (log_2 TPM) per gene represented. (**C**) Quantification of log_2 counts per million (CPM) of cyclophilin family member transcripts in Control, Restored, Restored + CsA Type D (n=3) and Type V (n=3) cells. Error bars represent mean ± s.d. (**D,E)** Comparison of p53 ChIP-seq signal between Restored and Restored + CsA Type V (n=3) cells for p53 hallmark genes (**d**) and Module 5 genes (**e**). Data are centered on p53-bound peaks across a ±1kb window. Source data are provided as a Source Data file.

Supplementary Figure 18

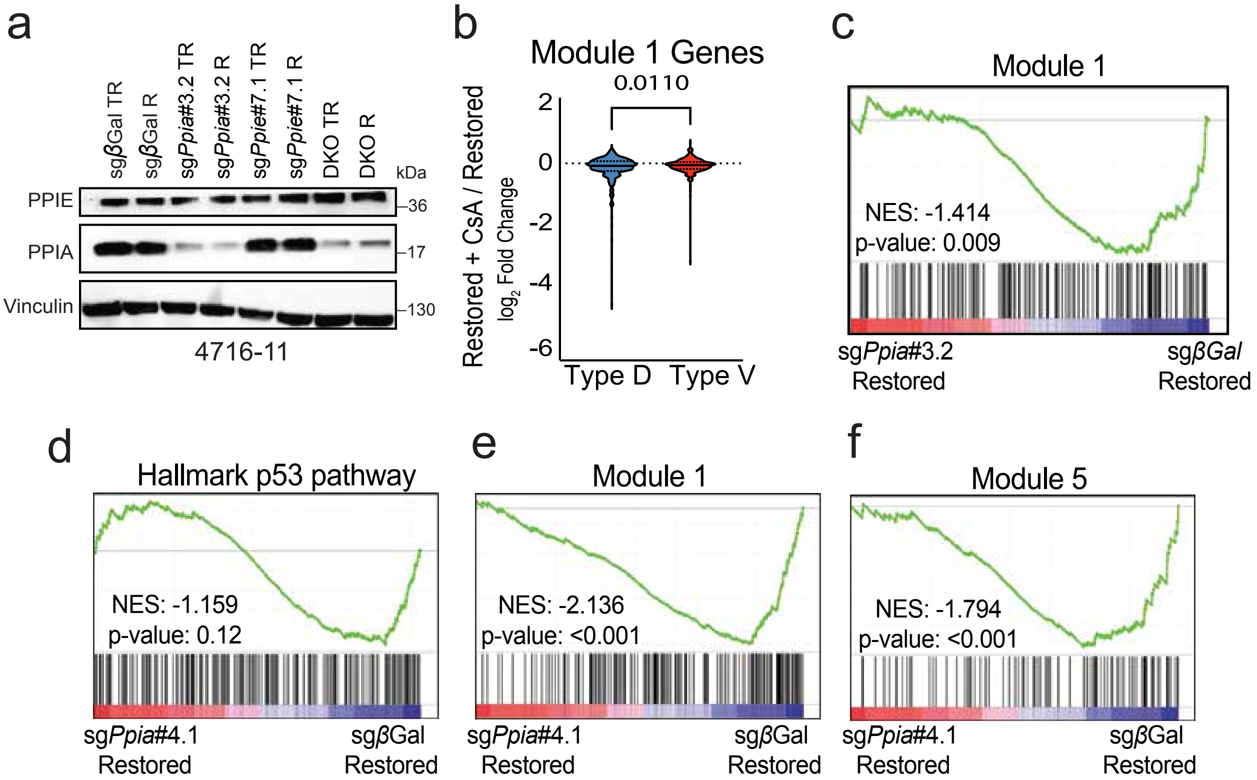

Supplementary Fig. 18. Cyclophilin A knockout decreases expression of p53-regulated genes in Type D cells. (A) Immunoblot analysis for cyclophilin A and cyclophilin E in Type D (4716-11) cell line expressing sgRNAs targeting β-Gal, *Ppia*, and/or *Ppie* 72 hours after 4-OHT treatment. Vinculin is loading control. Experiment was conducted at least 2 weeks after antibiotic selection and/or cell sorting of CRISPR-targeted cells. **(B)** Violin plots indicating $\log_2$ fold-change expression of Module 1 genes in Restored and Restored + CsA Type D (4711-1, 4711-18, 4716-11) and Type V (4711-11, 4716-10, 4716-14) cells. Statistical significance determined by two-tailed Student's *t*-test.(**C-F**) GSEA of Module 1 (**c**,**e**), "HALLMARK_P53_PATHWAY" (**d**), and Module 5 genes (**f**) in Type D (4711-1, 4711-18, 4716-11) cells expressing sgRNAs targeting β-Gal or exons 3 (**c**) and 4 (**d**,**e**,**f**) of the *Ppia* gene 72hrs after 4-OHT treatment. Nominal p-value calculated by permutation test using GSEA software. No adjustments were made since only one geneset was tested. Source data are provided as a Source Data file.

Supplementary Table 1: Mouse sgRNA sequences used in study

Target Gene	Sequence
sg*βgal*	CACGTAGATACGTCTGCATC
sg*Trp53*	AGGAGCTCCTGACACTCGGA
sg*Atg5#2*	TATCCCCTTTAGAATATATC
sg*Atg5#3*	AAGAGTCAGCTATTTGACGT
sg*Bax* #3.1	TCATCCAGGATCGAGCAGGG
sg*Bax* #4.1	CCCGGAAGAAGACCTCTCGG
sg*Bbc3* #2.1	ACCTCAACGCGCAGTACGAG
sg*Mlkl#2*	GACTTCATCAAAACGGCCCA
sg*Mlkl#3*	AACCCCCAGGCCGAAAGTGT
sg*Ripk1* #3.1	TGTGAAAGTCACGATCAACG
sg*Ripk1* #2.1	CAGACTGAGACACAGTCGAG
sg*Ppia* #3.1	TGGAATAATTCTGTGAAAGG
sg*Ppia* #3.2	GCTCTGAGCACTGGAGAGAA
sg*Ppia* #4.1	ACACGCCATAATGGCACTGG
sg*Ppib* #2.1	CAAATTGGAGATGAATCTGT
sg*Ppib* #3.1	TTCACCAGGGGAGATGGCAC
sg*Ppic* #5.1	ACTGTTGACGATGGTGCAGT
sg*Ppic* #3.1	TCATGAAGTCCTTGATGACA
sg*Ppid* #4.1	GGTCATAAAAGGACTAGGTG
sg*Ppid* #3.1	TCAAATCAGAATGGGACAGG
sg*Ppie* #10.1	TCACATGTACTCTCCACAGT
sg*Ppie* #7.1	TGGGCACCACATCAGACCGT
sg*Ppif* #1.1	TGGACAGCCGCTCGGCCGCG
sg*Ppif* #4.1	ACCACAATGGCACAGGAGGG
sg*Ppig* #3.1	TCTCTTTCATAGAGTTGTCA
sg*Ppig* #2.1	GCGAAAGTTCTCACACGTTT
sg*Ppih10* .1	CAATAAGCCCAAACTGCCAG
sg*Ppih* #9.1	GCTTGAACTCTCTTTCCCAA
