# Supplementary material for: p53 restoration in small cell lung cancer identifies a latent cyclophilin-dependent necrosis mechanism
Source: Nat Commun. 2023 Jul 21;14:4403. doi: 10.1038/s41467-023-40161-9 (PMC10362054; doi:10.1038/s41467-023-40161-9)
Supplement: Supplementary file 4 — Description of Additional Supplementary Files [file 41467_2023_40161_MOESM4_ESM.pdf]

DESCRIPTION OF ADDITIONAL SUPPLEMENTARY FILES

File name: Supplementary Movie 1

Description: p53 reactivation induces necrotic death in Type D tumor-derived SCLC cell lines. Live cell imaging of a Type D (4711-18) cell line. Images were acquired every 15 min for 4 days.

File name: Supplementary Movie 2

Description: p53 interacts with cyclophilin A in the nucleus. Movie of confocal 3D micrographs of p53-Cyp A interaction (red) using proximity ligation assay in Type D (4711-18) cells treated with 4-OHT for 48 hours. Nucleus is stained with DAPI (blue). Results were similar between the cross section and longitudinal section of the Type D cells. Scale bars, 10μm.

File name: Supplementary Data 1

Description: Differentially regulated genes in Type V and Type D SCLC cells.
